# Bitter, Sweet, Salty, Sour and Umami Taste Perception Decreases with Age: Sex-Specific Analysis, Modulation by Genetic Variants and Taste-Preference Associations in 18 to 80 Year-Old Subjects

**DOI:** 10.3390/nu10101539

**Published:** 2018-10-18

**Authors:** Rocio Barragán, Oscar Coltell, Olga Portolés, Eva M. Asensio, José V. Sorlí, Carolina Ortega-Azorín, José I. González, Carmen Sáiz, Rebeca Fernández-Carrión, Jose M. Ordovas, Dolores Corella

**Affiliations:** 1 CIBER Fisiopatología de la Obesidad y Nutrición, Instituto de Salud Carlos III, 28029 Madrid, Spain; rocio.barragan@uv.es (R.B.); oscar.coltell@uji.es (O.C.); Olga.Portoles@uv.es (O.P.); eva.m.asensio@uv.es (E.M.A.); jose.sorli@uv.es (J.V.S.); carolina.ortega@uv.es (C.O.-A.); Ignacio.Glez-Arraez@uv.es (J.I.G.); carmen.saiz@uv.es (C.S.), Rebeca.Fernandez@uv.es (R.F.-C.); 2 Department of Preventive Medicine and Public Health, School of Medicine, University of Valencia, 46010 Valencia, Spain; 3 Department of Computer Languages and Systems, Universitat Jaume I, 12071 Castellón, Spain; 4 Nutrition and Genomics Laboratory, JM-USDA Human Nutrition Research Center on Aging Tufts University, Boston, MA 02155, USA; jose.ordovas@tufts.edu; 5 Department of Cardiovascular Epidemiology and Population Genetics, Centro Nacional de Investigaciones Cardiovasculares (CNIC), 28029 Madrid, Spain; 6 IMDEA Alimentación, 28049 Madrid, Spain

**Keywords:** taste perception, aging, sex, taste polymorphisms, taste preferences

## Abstract

There is growing interest in relating taste perception to diet and healthy aging. However, there is still limited information on the influence of age, sex and genetics on taste acuity as well as on the relationship between taste perception and taste preferences. We have analysed the influence of age on the intensity rating of the five basic tastes: sweet, salty, bitter, sour and umami (separately and jointly in a “total taste score”) and their modulation by sex and genetics in a relatively healthy population (men and women) aged 18–80 years (*n* = 1020 Caucasian European participants). Taste perception was determined by challenging subjects with solutions of the five basic tastes using standard prototypical tastants (6-n-propylthiouracil (PROP), NaCl, sucrose, monopotassium glutamate and citric acid) at 5 increasing concentrations (I to V). We also measured taste preferences and determined the polymorphisms of the genes taste 2 receptor member 38 (TAS2R38), taste 1 receptor member 2 (TAS2R38) and sodium channel epithelial 1 beta subunit (SCNN1B), as TAS2R38-rs713598, TAS1R2-rs35874116 and SCNN1B-rs239345 respectively. We found a statistically significant decrease in taste perception (“total taste score”) with increasing age for all the concentrations analysed. This association was stronger for the higher concentrations (*p* = 0.028; *p* = 0.012; *p* = 0.005; *p* = 4.20 × 10^−5^ and *p* = 1.48 × 10^−7^, for I to V in the multivariable-adjusted models). When we analysed taste qualities (using concentration V), the intensity rating of all the 5 tastes was diminished with age (*p <* 0.05 for all). This inverse association differed depending on the test quality, being higher for bitter (PROP) and sour. Women perceived taste significantly more intense than men (*p* = 1.4 × 10^−8^ for total taste score). However, there were differences depending on the taste, umami being the lowest (*p* = 0.069). There was a complex association between the ability to perceive a taste and the preference for the same. Significant associations were, nevertheless, found between a higher perception of sour taste and a higher preference for it in women. In contrast, the higher perception of sweet was significantly associated with a higher preference for bitter in both, men and women. The TAS2R38-rs713598 was strongly associated with bitter (PROP) taste (*p* = 1.38 × 10^−50^), having a significant interaction with sex (*p* = 0.030). The TAS1R2-rs35874116 was not significantly associated with sweet, whereas the SCNN1B-rs239345 was associated (*p* = 0.040) with salty taste. In conclusion, the inverse association between age and perceived taste intensity as well as the additional influence of sex and some genetic polymorphisms give rise to large inter-individual differences in taste perception and taste preferences that should be taken into account in future studies and for applications in precision nutrition for healthy aging.

## 1. Introduction

The elderly population is increasing worldwide [1], as people now live longer than ever before as a result of medical and social progress [1,2]. Although this increase in life expectancy is an important achievement, it is crucial to combine it with improvements in quality of life and other indicators such as so-called “Healthy Life Years” [3,4]. Therefore, knowledge of the lifestyle factors that contribute to people having a better quality of life it should be one of the main priorities of health systems [3,5,6]. Diet is one of such lifestyle factors that could have the greatest impact on health and quality of life [7,8,9,10]. Currently, new evidence is emerging on the importance of making more specific nutritional recommendations for each person or group of people depending on their characteristics and needs [11]. More attention, therefore, is now being paid to the nutritional needs related to healthy aging [12], whether that be more specifically attending to the deficiencies or excesses that the elderly present [13,14,15], or designing strategies earlier for younger people so that diet can help to delay aging by acting on the telomere length, on deoxyribonucleic acid (DNA) methylation or other measurements of biological aging [16,17,18,19].

However, another additional factor that is seldom taken into account is that not only is it important to make dietary recommendations on the best composition of the diet to achieve healthy aging but to also study the factors that contribute to their adherence to such recommendations [20,21]. In general, people prefer to eat foods they like [21,22,23] and taste is one of the most important factors when making the choices [21,22,24,25]. Although there is huge inter-individual variation in the perception of the five basic tastes (sweet, salty, bitter, sour and umami) [26,27,28], little is known about the influence of such variation on the intake patterns of certain foods [29,30] or even on preferences for certain tastes [26,27,31,32], emphasizing the need for further research. Initial findings in humans and in animal models suggest that lower taste perception may be associated with higher obesity risk [33,34,35]. Moreover, aging is associated with a decrease in all senses [36]. However, whereas sight is frequently measured and its decrease corrected, this does not happen with taste, given that it is not routinely measured and neither is its correction considered [37]. Hence, the influence of the decrease in taste perception in general, or a decrease in specific tastes, in particular, on either diet or health is not well known and recent review concerning the influence of aging on the perception of the different tastes, food preferences and diet, put in evidence the scarcity of relevant studies and the methodological limitations associated with the existing studies [38]. In addition to age, sex is another relevant variable that has been poorly analysed regarding taste perception and that requires more attention in the new era for precision nutrition [11]. Although Fisher, et al. [28] in a large epidemiological study reported statistically significant differences between men and women for bitter, sweet, salty and sour tastes, in other studies differences by sex were not analysed [26,27]. Taste has a genetic component, although most of our knowledge relates to bitter taste [39,40,41]. Thus, polymorphisms in the taste 2 receptor member 38 (TAS2R38) gene have been closely associated with greater bitter taste perception of the tastants phenylthiocarbamide (PTC) and 6-n-propylthiouracil (PROP) among different populations [42,43,44]. Specifically, the rs713598 polymorphism in the TAS2R38 gene (TAS2R38-rs713598) consisting of a Ala49Pro amino acid change has been selected as the tag single nucleotide polymorphism (SNP) in several studies [26,43,45]. Although for sweet and salty tastes, there is much less consistency than for bitter taste, several polymorphisms have been identified in genes related to these tastes in association with different phenotypes [27,30,46,47,48]. Among them, both the rs35874116 polymorphism in the taste 1 receptor member 2 (TAS2R38) gene (TAS1R2-rs35874116) and the rs239345 polymorphism in the sodium channel epithelial 1 beta subunit (SCNN1B) gene (SCNN1B-rs239345) for the sweet and salty tastes, respectively, have been analysed with inconsistent results [47,48,49,50,51]. Thus, there is a need to incorporate taste-related genetic factors into large studies that analyse the relationship between age and taste perception and modulation by sex. Given that, at present, no study has been published that has widely analysed all these factors in determining taste perception of five tastes in the same population with a wide age range and relatively large sample size, the aims of our study are as follows: (1) To analyse the association between the perception of the five basic tastes (separately and jointly) and age, both in the total sample and in men and women; (2) To analyse the association between taste perception and preferences for different tastes, both generally and per age and sex groups; and (3) To study the genetic influence (of three relevant polymorphisms in genes related with bitter (PROP), sweet and salty tastes) on taste perception in this population and its heterogeneity per sex and age.

## 2. Materials and Methods

### 2.1. Study Design and Participants

We have carried out a cross-sectional analysis on 1020 Caucasian participants (365 men and 655 women) between the ages of 18 and 80 years old recruited in the Obesity, Nutrition & Information and Communication Technologies (OBENUTIC) study. OBENUTIC [52] is a case-control study carried out in the general population of the Valencia Region (East Mediterranean coast of Spain). Cases were individuals with obesity (body mass inde × (BMI) ≥ 30 kg/m^2^) and the controls were non-obese individuals (BMI < 30 kg/m^2^) recruited from the same location and without pairing for age and sex. Cases and controls were apparently healthy individuals recruited through advertisements in shopping malls, housewives’ associations, cultural associations and other types of groups from the general population, public and private institutions, educational centres, home contacts and a few primary health care centres. The exclusion criteria were being pregnant or breast-feeding, suffering from some type of infectious/contagious disease, invalidating physical or psychological diseases, cancer diagnosis, thyroid alterations, Cushing disease, have conditions that could alter gustatory functions (e.g., tooth extraction), high alcohol intake or the consumption of other drugs.

Participants visited the Genetic and Molecular Epidemiology Unit and the sensory research laboratory at Department of Preventive Medicine and Public Health at the University of Valencia, Valencia, on two-three separate sessions within a week. At the first session, participants completed a health and demographic questionnaire and their anthropometric parameters and blood pressure were measured as indicated. Participants also completed lifestyle questionnaires and the food preference questionnaires and were scheduled for a blood venepuncture in fasting conditions for biochemical analysis and DNA isolation. In addition, participants were scheduled for the taste perception tests under standardized conditions. Initially, the OBENUTIC study began in 2007 by only including the bitter taste perception tests but later, the project was completed by including the perception tests for the other tastes. In this study, we analysed 1020 participants (365 men and 655 women) for those who consecutively had the complete data available for the perception tests of bitter, sweet, sour, salty and umami tastes. Participants provided written informed consent and study protocol and procedures were approved according to the ethical standards of the Helsinki Declaration and by the Human Research Ethics Committee of the University of Valencia, Valencia.

### 2.2. Demographic, Anthropometric, Biochemical, Clinical and Lifestyle Variables

Socio-demographic and clinical variables were obtained through a standardized questionnaire previously used in our studies [53]. Anthropometric variables and blood pressure were determined by trained staff and in accordance with the standard recommendations. Weight and height were directly measured with calibrated scales (TANITA-BC-420-S, Tanita UK Ltd., Middlesex, UK) and a standard, calibrated stadiometer (SECA Mod 220, Seca Deutschland Gmbh & Co. Kg., Hamburg, Germany), incorporated into the scales, respectively. Body mass index (BMI) was calculated as the weight in kilograms divided by height in square meters. Waist circumference was measured midway between the lowest rib and the iliac crest, after normal exhalation, using an anthropometric tape. Blood pressure was measured with the use of a validated semiautomatic oscillometer (Omron HEM-705CP, OMRON Healthcare Europe B.V., Hoofddorp, The Netherlands) while the participant was in a seated position for 5 min. Blood samples were collected after a 12-hour overnight fast. Fasting plasma glucose, HDL-C and triglyceride concentrations were measured as previously described [53] and LDL-C was estimated by the Friedewald equation. Clinical variables (personal and family history of disease) as well as medication use were assessed by questionnaire. Diabetes was defined as previous clinical diagnosis of diabetes. For tobacco smoking, subjects were classified as current or non-current smokers.

### 2.3. Taste Preference Assessment

Besides the taste perception laboratory tests, the participants had to complete another questionnaire on their hedonic judgment rating the preference for the different tastes. Thus, the participants had to rate on a quantitative scale from zero (minimum; extremely dislike) to 3 (maximum; extremely like: favourite taste) their preference for each taste. Only questions on preferences for sweet, salty, sour and bitter were included.

### 2.4. Taste Perception Tests

Taste perception test were carried out on the 1020 participants using the same methodology, standardized for that purpose. Tests were carried out in the morning in our laboratory, suited to that purpose in an appropriate temperature, comfort and silence so as to allow good concentration when undertaking the test. Trained staff provided a detailed explanation of the procedures prior to starting the series of tests. For each taste quality, a representative compound was chosen, which was administered at five concentrations. In one test session, the participants were subjected to the tasting of the five concentrations of five representative tastant for bitter, sweet, salty, sour and umami tastes. For each taste, the following tastants were used (all from Sigma-Aldrich, Milan, Italy): PROP, sucrose, NaCl, citric acid and L-glutamic acid monopotassium salt monohydrate (MPG), for bitter (PROP), sweet, salty, sour and umami tastes, respectively. Distilled water was used as the solvent. Each tastant was presented to subjects independently. In addition, the participants were tested for their perception of bitter taste using phenylthiocarbamide (PTC); however, these data have not been analysed in the current study (although historically PTC was the first compound used to test the degree of bitter taste perception, this compound was considered to present several drawbacks, among them that it had a certain smell and toxicity, so PROP was introduced as an alternative tastant for bitter taste but both PTC and PROP are genetically determined by similar loci). The various solutions of different concentration were prepared for each tastant by trained personnel, also including a distilled-water control. The series of concentrations (concentrations I, II, III, IV and V, respectively) used for each tastant was based on previous reports [54,55,56,57,58] and were as follows: for PROP (0.055, 0.17, 0.55, 1.7 and 5.5 mM); for sucrose (100 mM, 150 mM, 200 mM, 300 mM and 400 mM), for NaCl (25 mM, 50 mM, 75 mM, 100 mM and 200 mM); for citric acid (1 mM, 5 mM, 10 mM, 17mM and 34 mM); and for MPG (25 mM, 50 mM, 75 mM, 100 mM and 200 mM).

Bitter taste perception tests (PROP), were undertaken on strips of filter paper [54]. Filter papers were prepared by dipping Whatman no. 1 paper into the corresponding solution heated close to boiling point, dried and cut into strips and stored in glassine envelopes. The other tastants were prepared and tested in liquid form dissolved to the concentration indicated and were presented in different coloured small tubes for each taste, labelled with symbols and organized into racks of a pre-set order and the same for all participants. Before beginning the taste perception tests, participants had to rinse their mouths several times with spring water. All participants were given a template on which they had to complete a scale of taste perception intensity rating for each taste and concentration. The scale consisted of 6 intensity values (from 0 to 5), 0 meaning “no taste” and 5 “extremely strong”. Each subject was asked to place drops of the solution for a few seconds or the filter paper with the tastant on the tip of the tongue and then spit it out and rate the taste. Subjects rated the corresponding tastant solution selecting in the corresponding template the score that most closely approximated their sensation magnitude. The mouth was rinsed with water before and after each tastant challenge. Between each set of major taste challenges there was a 3–5 min break. The same scoring scale was used for all tastes, whether for bitter taste on filter paper, or for the other tastes in liquid solution. With the scores for the individual taste qualities, we constructed a “total taste score” for each concentration (total taste score for concentration I, total taste score for concentration II, total taste scores for concentration III, total taste score for concentration IV and total taste score for concentration V), summing up the points obtained for each of the individual tastes for each concentration tested (concentrations I to V). The range of the total taste score for each concentration was from 0 to 25 points. Total taste scores for concentrations I to V were used as a combined measure of global taste perception as previously used in some studies [33] to analyse the association between the global taste perception and age. However, for individual tastes, the association analyses with age, sex and genetic polymorphisms were focused on the highest concentration (concentration V) analysed (5.5 mM for PROP; 400 mM for sucrose; 200 mM for NaCl and MPG; and 34 mM for citric acid) in order to maximize the differences in intensity rating between individuals.

### 2.5. DNA Genotyping

Genomic DNA was isolated from white blood cells. The TAS2R38-rs713598, TAS1R2-rs35874116 and SCNN1B-rs239345 polymorphisms were determined in the same laboratory using an ABI Prism 7900HT Sequence Detection system (Applied Biosystems by Life Technologies) (Thermo Fisher Scientific Inc., Waltham, MA, USA) and the corresponding fluorescent allelic discrimination TaqMan assays by standard procedures in subjects with DNA available. For quality control purposes, 10% of randomly selected samples were genotyped a second time and there were no discrepancies. Genotyping was carried out on all participants whose DNA was available. Finally, 945 for the TAS2R38-rs713598 SNP, 949 valid genotypes for TAS1R2-rs35874116 SNP and 927 for the SCNN1B-rs239345 SNP were obtained. All polymorphisms were in Hardy–Weinberg equilibrium (*p* = 0.998; *p* = 0.549 and *p* = 0.770 for the TAS2R38-rs713598, TAS1R2-rs35874116 and SCNN1B-rs239345, respectively).

### 2.6. Statistical Analysis

Analyses were undertaken on the entire sample studied and also stratified by sex. Chi-square tests were used to compare proportions. Student *t* tests and ANOVA tests were applied to compare crude means of continuous variables. In addition, the non-parametric Mann-Whitney *U* Test or the Kruskal–Wallis were used to compare means when indicated. Triglyceride concentrations were log-transformed for the statistical analyses. Age was used as a continuous variable and also a categorical variable was created with three age groups. The three age groups were created taking into account the population tertiles, as follows: from 18 to 36 years, from 37 to 50 years and from 51 to 80 years. The genotype variables were initially used as three categories and later the additive, dominant and recessive models were tested using the model which better fitted the data for each polymorphism. Correlation coefficients (Spearman rho) between taste perception for the different tastants in the whole population and by gender and age were estimated. Also, Spearman rho coefficients were calculated to analyse the correlation between taste preference and taste perception in the whole population and by sex and age groups. Crude models of association between taste perception and anthropometric variables were adjusted for potential confounders by multivariable adjusted regression models. General linear models were used to test the association between taste perception (considered as continuous variables) and the predictors. Models were sequentially adjusted as follows: model 1, unadjusted; model 2, adjusted for age and sex; model 3, additionally adjusted for other covariates such as BMI/case-control, type-2 diabetes, smoking and medications (lipid lowering drugs and antihypertensive drugs). Adjusted means were estimated, both generally and stratified by sex, age group or genotype when indicated. Depending on the type of analysis undertaken, some models included a later adjustment for other additional variables, among them, the genetic polymorphisms, details of which are provided for each specific model. To test for heterogeneity per sex, age or genetic polymorphism hierarchical multivariate models were used including the main variables and the corresponding interaction terms. Statistical analyses were performed with the IBM SPSS Statistics for Windows, Version 24.0 (IBM Corp., Armonk, NY, USA) All tests were two-tailed and *p*-values < 0.05 were considered statistically significant for these associations.

## 3. Results

### 3.1. General Characteristics of the Population

Table 1 presents the baseline demographic, clinical, lifestyle and genetic characteristics of the study participants by sex. Of the 1020 participants studied, 364 were men and 655 women. The mean overall age was 43.2 years, without statistically significant differences between men and women (*p* = 0.095). The age range was from 18 to 80 years old. Later a variable was created with three age categories depending on tertiles of population (18–36 years (*n* = 342), 37–50 years (*n* = 329) and 51–80 years (*n* = 349)) in order to present the results per age group. 281 participants were obese cases and 739 were non-obese. This was a relatively healthy population with low prevalence of diabetes (4.5% in the whole population) and without statistically significant differences (*p* = 0.423) between men and women. Plasma lipid concentrations were also within the normality range. A small percentage of the population was reported to be taking drugs for blood pressure (12.9%) or for lipids (12.1%). Table 1 also presents the prevalence of the three polymorphisms analysed including the bitter (PROP) gene (TAS2R38), the sweet gene (TAS1R2) and the salty gene (SCNN1B).

### 3.2. Descriptive of the Taste Perception Tests

For each taste, 5 increasing concentrations (I, II, III, IV and V) of the representative tastants were tested (see Methods). Figure S1 shows ratings (means and SE) of perceived taste intensity in response to different tastant concentrations for bitter (PROP), sweet, salty, sour and umami tastes for each of the concentrations tested, scored on a scale from 0 to 5 in the whole population (model 1). As the tastant concentration increased, the rating score for all of them also increased. With these scores, we constructed the “total taste score” (from I to V), summing up the points obtained for each of the individual tastes for each concentration tested (concentrations I to V). In total, the range of each total taste score was from 0 to 25 points for each concentration. The total taste scores (mean and SD) for the 5 concentrations in the whole population (unadjusted date) were as follows: 3.16 (2.41), 4.62 (2.84), 6.53 (3.51), 8.22 (3.99) and 11.22 (4.69) points for concentrations I, II, III, IV and V, respectively. Figure S2 shows frequency distribution of the total taste score for concentration V in the whole population.

### 3.3. Associations between Age and Total Taste Score for the Different Concentrations of Tastants (I to V)

**Figure 1:**
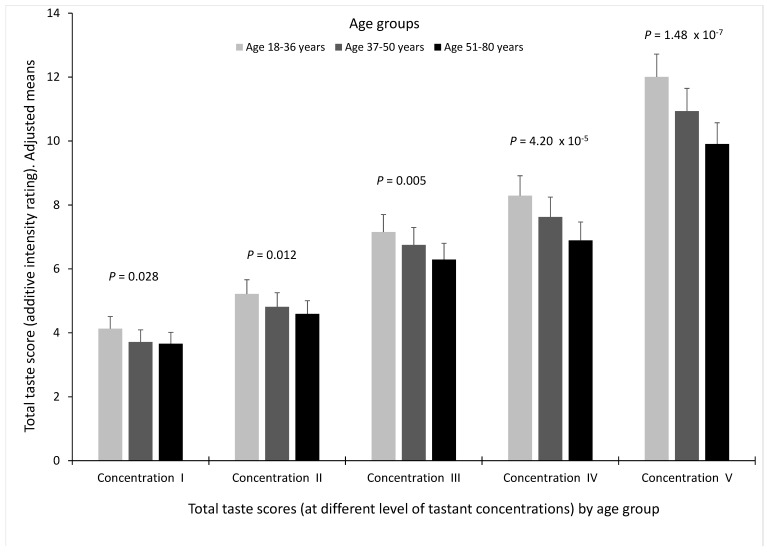
Total taste score (sum of intensity ratings of the five tastes) by age group and the increasing concentrations (from I to V) of the tastants tested (*n* = 1019, 1016, 1014, 1016 and 1020 for the concentrations I, II, III, IV and V), respectively in the whole population). Values are adjusted means (model 3) including sex, age, diabetes, body mass index, smoking and medications. Age groups are based on tertiles. *p*-values show the statistical significance for the age differences in total taste perception for each concentration after adjustment for covariates (model 3). Error bars are SE. Age groups: 18–36 years (*n* = 342), 37–50 years (*n* = 329) and 51–80 years (*n* = 349).

Total taste scores for concentrations I to V were used to analyse taste perception by age. Figure 1 shows total taste score of the five tastes by age group and the increasing concentrations in the whole population.

In the multivariate adjusted model for sex, diabetes, BMI, smoking and medications, we found a statistically significant decrease in total taste perception with increasing age for all the concentrations analysed (younger participants perceived stronger intensities, while older participants perceived weaker intensities for the same tastant concentration). However, this association was higher for the higher concentrations of the tastants tested (*p* = 1.48 × 10^−7^ for concentration V; *p* = 4.20 × 10^−5^ for concentration IV, *p* = 0.005 for concentration III, *p* = 0.012 for concentration II and *p* = 0.028 for concentration I). Given that in the highest concentration for each taste (concentration V) is where the highest intensity scores are obtained and for the other concentrations, the differences by age are minimized, only the highest concentration of each tastant (concentration V) was used for the main analyses of this work as a measurement of the intensity of each taste perception.

### 3.4. Correlation between Perception of the Different Tastes and Association between Age and the Five Tastes at Concentration V

Table 2 presents unadjusted mean for each tastant and for the total taste score as well as correlation coefficients between tastants in the whole population. Table S1 presents these analyses in men and women. The highest correlation coefficient (positive) among individual tastants was for sour and salty tastes (*r* = 0.569; *p <* 0.001 in the whole population), this result being highly consistent in both men (*r* = 0.536; *p <* 0.001) and women (*r* = 0.571; *p <* 0.001). The weakest correlation was between bitter (PROP) and sweet tastes, also for both sexes. The total taste score has a high positive correlation coefficient with all the tastes, particularly salty (*r* = 0.770; *p <* 0.001 in the whole population).

**Figure 2:**
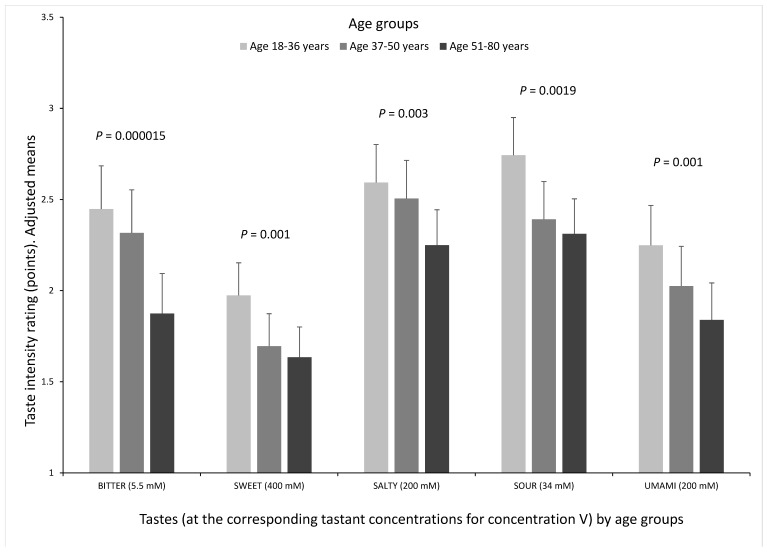
Intensity rating (points) for the five basic tastes (bitter (PROP), sweet, salty, sour and umami) by age group (18–36 years (*n* = 342), 37–50 years (*n* = 329) and 51–80 years (*n* = 349)) at the maximum concentration (concentration V) of the tastants tested (PROP 5.5 mM; sucrose 400 mM, NaCl 200 mM; citric acid 34 mM; and MPG 200 mM), in the whole population (*n* = 1020). Values are adjusted means (model 3) including sex, age, diabetes, body mass index, smoking and medications. Age groups are based on tertiles. *p*-values show the statistical significance for the age differences in taste perception among groups for each taste after adjustment for covariates (model 3). Error bars are SE.

In the whole population (Table 3) we found a highly significant association between age (as continuous in years) and a lower taste perception for all tastes and for their sum (B = −0.070 (0.029) points/year age; *p* = 2.4 × 10^−9^) even in the multivariate model (model 3) adjusted for sex, BMI, diabetes, smoking and medications. The inverse association between age and taste perception intensity was different dependent on the test quality. The greatest decrease with age was observed for bitter (PROP) (*p* = 7.8 × 10^−7^) and sour (*p* = 6.0 × 10^−6^) tastes in the multivariate adjusted model 3. Figure 2 depicts the taste perception analysis by age groups to better visualize the associations and differences among tastes.

### 3.5. Influence of Sex in Taste Perception

**Figure 3:**
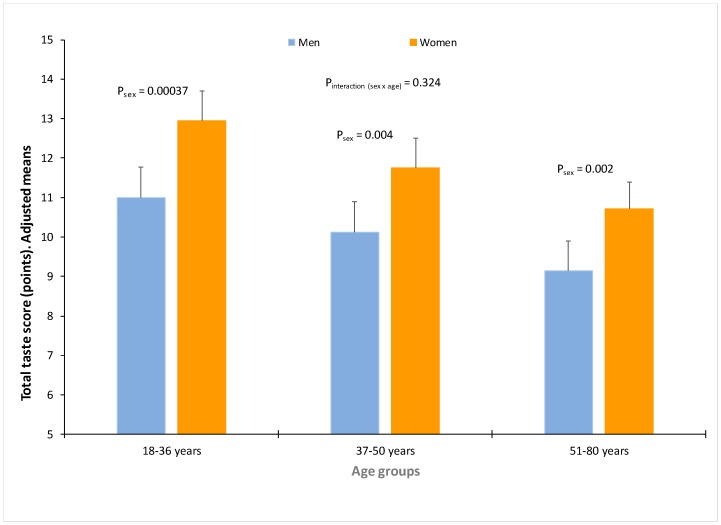
Total taste score (sum of intensity ratings of the five tastes) at the maximum concentration of the tastants tested (concentration V) by age group (18–36 years (*n* = 342), 37–50 years (*n* = 329) and 51–80 years (*n* = 349)) in men (*n* = 365) and women (*n* = 655). Values are adjusted means (model 3) including sex, age, diabetes, body mass index, smoking and medications. *p*-values show the statistical significance of the differences in total taste perception by sex in each age group, after adjustment for covariates (model 3). Error bars are SE.

For the total taste score (concentration V) women perceived taste significantly more intense than men (B = 1.720 (0.301) points higher for women in comparison with men; *p* = 1.4 × 10^−8^) after multivariate adjustment (model 3) for age, BMI, diabetes, smoking and medications (Table 4). However, there were differences in the intensity rating by sex depending on the taste. Differences by sex were higher in women for sour (1.6 × 10^−8^), bitter (PROP) (1.3 × 10^−6^) and salty (1.3 × 10^−6^). Non-statistically significant differences by sex were found for umami rating (*p* = 0.069). When we analysed the homogeneity or heterogeneity of the sex influence by age, we did not obtain statistically significant interactions terms (Table 4) but for some tastes (umami and sweet), the *p*-value for the interaction terms was close to 0.1 suggesting some potential heterogeneity. We explored the potential heterogeneity per age in the sex effects, and, for the total taste score (Figure 3), although in all age groups, perceived intensities were strongest for women than for men, these differences tended to be higher in the youngest age group. For bitter (PROP) taste (Figure S3), there was a strong homogeneity on the sex differences by age. However, for umami, the sex effect (although no significant), tended to be higher in the youngest group and was cancelled in the older.

### 3.6. Taste Preferences and Association with Taste Perception

In the whole population, participants liked the sweet taste most followed by salty. Bitter and sour were the most disliked (Table 5). No statistically significant differences by sex were detected for sweet and salty. However, men liked the bitter (PROP) and the sour tastes more than women (*p <* 0.001 and *p* = 0.007, respectively). By age groups we found some statistically significant differences in taste preferences (Table S2). The older group reported lower liking ratings for bitter (PROP) (*p <* 0.001) and for sweet (*p* = 0.004) tastes compared with the young participants. The liking ratings for sour and salty tastes did not change across age-groups. Regarding the associations between preferences for different tastes (Table 5 and Table S2), in the whole population we observed a strong inverse association between liking for sweet and liking for and bitter (PROP) taste (*r* = −0.175 and *p* < 0.001). This result was highly consistent in both men (*r* = −0.181; *p <* 0.001) and women (*r* = −0.181; *p <* 0.001) and across age groups (Table S2). In the whole population, sour liking was positively associated with bitter (PROP) and salty liking (*r* = 0.346; *p <* 0.001 and *r* = 0.131; *p* < 0.001, respectively) and negatively related to sweet taste liking (*r* = −0.125; *p <* 0.001). With few exceptions, these correlations were similar by sex and age groups (Table 5 and Table S2).

Further, we analysed the association between taste preferences and taste intensity rating (concentration V). For the whole population (Table S3), we observed that in general, a higher intensity rating for a particular taste was not associated with a higher preference for this taste. Interestingly, we observed positive correlations with some of the opposite tastes. Thus, a greater acuity for sweet taste was significantly associated with a higher preference for bitter (PROP) (*r* = 0.101; *p* = 0.002) and sour (*r* = 0.102; *p* = 0.002) tastes.

These results were very consistent in both men and women (Table 6). In the analysis by sex, in women, dislike of the salty taste was determined by its perceived intensity (*r* = −0.103; *p* = 0.011). In contrast, a higher perception of the sour taste was associated with a higher preference for this taste in women (*r* = 0.085; *p* = 0.036).

### 3.7. Association between Genetic Polymorphisms and Taste Perception

**Figure 4:**
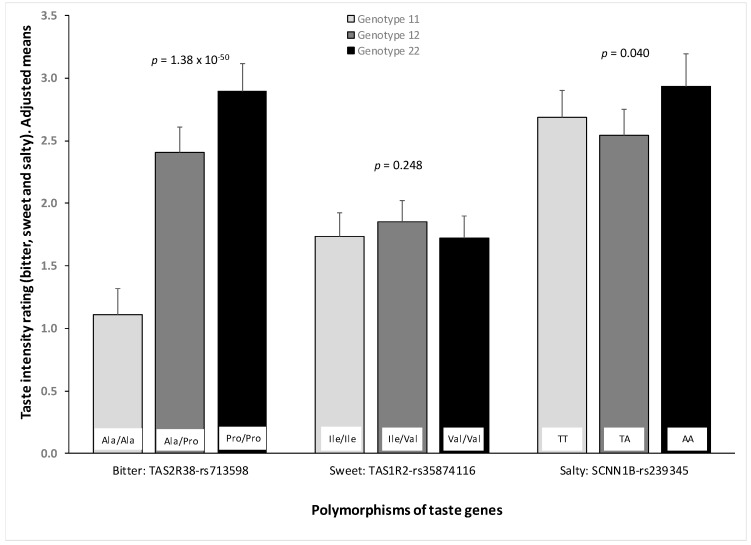
Taste perception (intensity rating) of bitter (PROP, 5.5 mM), sweet (sucrose, 400 mM) and salty (NaCl, 200 mM) in the whole population depending on the genotype for the corresponding taste polymorphism: TAS2R38-rs713598 for bitter (PROP); TAS1R2-rs35874116 for sweet; and SCNN1B-rs239345 for salty. Means were adjusted for sex, age, diabetes, body mass index, smoking and medications (model 3). *p*-values show the statistical significance for the corresponding taste polymorphism (3 genotypes as codominant) in the multivariate adjusted model 3. *n* = 945 for TAS2R38-rs713598; *n* = 949 for TAS1R2-rs35874116; and *n* = 927 for SCNN1B-rs239345. Error bars are SE.

Figure 4 shows the association between the intensity rating for bitter (PROP), sweet and salty tastes and the selected polymorphisms in each gene: TAS2R38-rs713598, TAS1R2-rs35874116 and SCNN1B-rs239345, respectively. Models were adjusted for age, sex, BMI, diabetes, smoking and medications. As expected, we observed a robust association between the TAS2R38-rs713598 polymorphism and bitter (PROP) taste (*p* = 1.38 × 10^−50^), with Ala/Ala subjects having a low perception of the bitter (PROP) taste. However, for the sweet taste polymorphism, we did not find any significant association (neither for the co-dominant model, nor for the dominant or recessive models). For the salty polymorphism, we detected a weak significant association in the co-dominant model (*p* = 0.040). This association tended to follow a recessive model with homozygous subjects for the minor allele having higher levels of salty taste perception (*p <* 0.05). When we analysed the influence of the polymorphisms on the age effect of decreasing taste perception, we did not find statistically significant interaction terms for any of the polymorphisms analysed.

**Figure 5:**
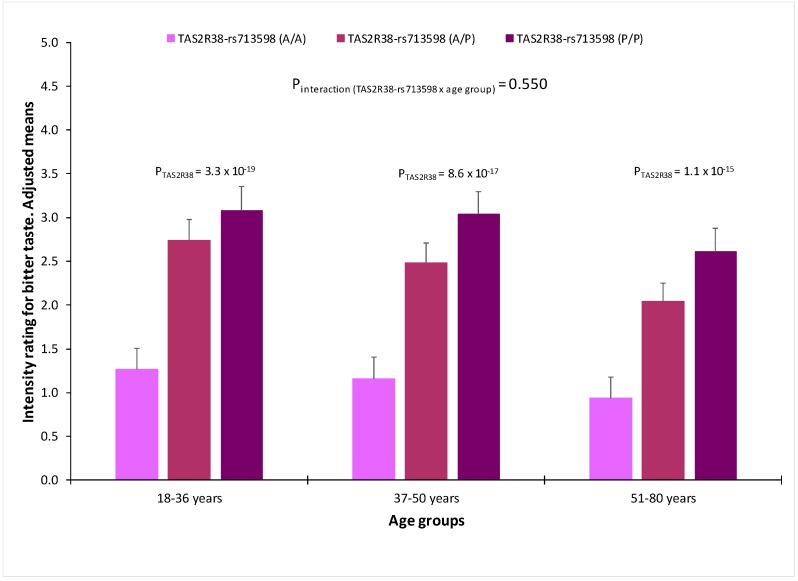
Bitter taste perception (intensity rating of PROP, 5.5 mM) in the whole population (*n* = 949) by the TAS2R38-rs713598 polymorphism (*n* = 304 for Ala/Ala, *n* = 464 for Ala/Pro and *n* = 177 for Pro/Pro) and age group. Means were adjusted for sex, age, diabetes, body mass index, smoking and medications (model 3). *p*-values show the statistical significance of the TAS2R38-rs713598 polymorphism, in each age group, in the multivariate adjusted model 3. The *p*-value for interaction term between the TAS2R38-rs713598 and age groups was additionally tested in model 3. Error bars are SE.

**Figure 6:**
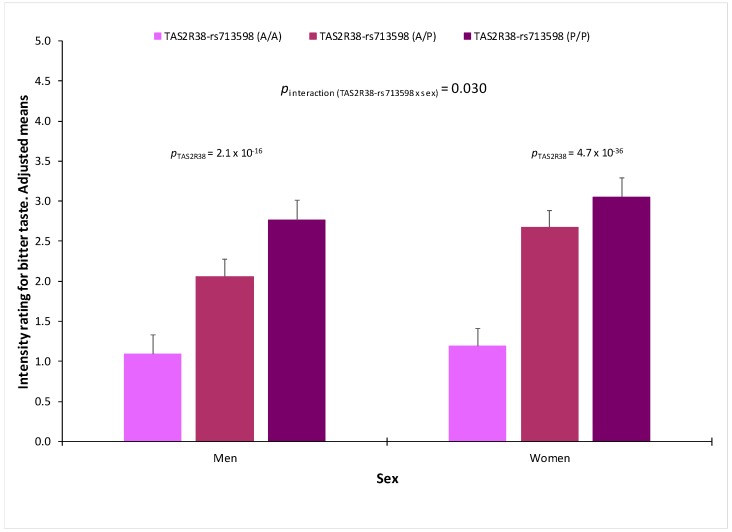
Bitter taste perception (intensity rating of PROP, 5.5 mM) in men (*n* = 342) and women (*n* = 603) by the TAS2R38-rs713598 polymorphism (men: *n* = 116 for Ala/Ala, *n* = 157 for Ala/Pro and *n* = 69 for Pro/Pro; women: *n* = 188 for Ala/Ala, *n* = 307 for Ala/Pro and *n* = 108 for Pro/Pro). Means were adjusted for sex, age, diabetes, body mass index, smoking and medications (model 3). *p*-values show the statistical significance of the TAS2R38-rs713598 polymorphism stratified by sex, in the multivariate adjusted model 3. The *p*-value for interaction term between the TAS2R38-rs713598 and sex was additionally tested in model 3. Error bars are SE.

Figure 5 shows bitter (PROP) taste perception in the whole population by the TAS2R38-rs713598 polymorphism in the three age groups. In all the age groups, the effect of the genetic polymorphism was highly statistically significant and the interaction term between the polymorphism and age did not reach the statistical significance (*p* = 0.550 in the multivariate adjusted model). However, when we analysed the influence of sex (Figure 6), we detected a statistically significant interaction term (*p* = 0.030) between the TAS2R38-rs713598 polymorphism and sex in determining bitter (PROP) taste perception. Thus, in homozygous for the non-taster polymorphism (Ala/Ala), there were no sex differences in the perceived intensity (1.10+/−0.23 points in men versus 1.19+/−0.22 points in women; *p* = 0.714). For sweet (results not shown) and for salty (Figure S4), no significant gene-sex or gene-age interactions on the corresponding perceived intensity were detected.

## 4. Discussion

This study on a general, relatively healthy, European population between the ages of 18 and 80, shows a significant decrease in the perception of different tastes with increasing age. The decrease of the intensity of taste perception was statistically significant for all of them (bitter, sweet, salty, sour and umami), as well as for their sum, which we have called “total taste score.” This inverse association by age differed depending on the test quality tested, being greater for bitter (PROP) and sour. Although the decrease in taste perception with aging has been investigated for a long time [38,58], no previous study has been published that has analysed the perception of all basic tastes and their sum using the same methodology with various concentrations of representative tastants in a wide sample of a relatively healthy general population between the ages of 18 and 80 years controlling for potential confounder variables, as well as studying the interactions per sex, age group or genetic polymorphisms. In the review undertaken on this topic by Sergi, et al. [58], the authors concluded that there was only sufficient evidence to conclude an age-related decrease in the perception of bitter (PROP) and sour tastes and that more studies were required to clearly conclude the decrease in the perception of other tastes. Our study, therefore, is useful in that it provides more specific data on quantification and better characterization of the decrease in the perception of the different tastes with age; other studies have analysed fewer taste qualities [59,60,61,62,63,64,65] have focused on a shorter age range [64,65], have studied children and their mothers [65]; or when the five taste qualities have been studied, their sample size has been very small [38,58,66]. Therefore, the results of these individual studies regarding the association between taste perception qualities and age have been heterogeneous. Among them, the Beaver Dam Offspring Study (BOSS) [28], undertaken in Wisconsin on a large sample of participants (*n* = 2374) with ages ranging from 21 to 84 years old, did not even measure the 5 tastes but only 4 of them (sweet, salty, bitter and sour) and only used one concentration for each tastant. In this study, the authors only found a statistically significant association between age and sweet taste perception [28]. In the BOSS study, the tastant used for bitter taste perception was quinine [28]. Although in our study we used PROP to test the ability to perceive bitter taste, being the compound traditionally used as representative of this taste (and similar to PTC), there are also other tastants to detect bitter taste perception, among them quinine (used in the BOSS study) and caffeine [67]. More studies in large samples are required using other tastants of bitter taste perception in order to characterize more fully how the perception of the different bitter compounds varies with age. However, in agreement with our results, Hansen, et al. [67] in Australia also reported an inverse association between age and the bitter taste of caffeine as well as of PTC. In addition to age, sex is another factor for which the results of the different studies carried out have been heterogeneous. In the BOSS study [28], they found statistically significant associations with sex for the 4 tastes analysed (bitter, sweet, salty and sour), in such a way that women perceived these tastes more than men. In our study, we also found that women consistently have a greater perception of 4 (bitter, sweet, salty and sour) of the 5 tastes analysed. No statistically significant differences by sex were detected for the umami taste. In general, a greater taste perception in women has also been reported for several taste qualities in other studies [43,58,60,61,62,63,68] although the results are not always consistent. That may be due to differences between populations, to a deficient control for age or for other possible confounding variables, to the tastant concentration, to the methodology with which the taste perception test was carried out, to the lack of statistical power by analysing small sample sizes or even to the ethnic differences [69]. In our study, not only have we analysed the influence of sex on taste perception but also the homogeneity/heterogeneity of that influence on the different ages by testing the statistical significance of the interaction term between sex and age on determining perceived intensities. In general, this analysis did not find any statistically significant interaction term between sex and age, so we may conclude that in the age range analysed, women perceive taste more than men in all age groups. However, the fact that for some tastes such as umami, the interaction term between sex and age was on the limit of statistical significance, so suggesting that the greater perception of women for this taste occurs in the youngest age group and diminishes in the elderly, means that further studies are required with larger sample size in order to investigate better this potential heterogeneity.

In our study, we also analysed the influence of genetic polymorphisms on selected relevant genes for bitter (PROP), sweet and salty and the perception of their corresponding taste. The genetics of taste perception is still little known and currently only the polymorphisms in the TAS2R38 gene are consistently associated with significant differences in bitter (PROP) taste perception in several populations [26,42,43,44]. For caffeine and quinine as bitter tastants, other loci have been identified [67]. Here, we selected as tag SNP the TAS2R38-rs713598 consisting of a A49P change which, as might be expected, was strongly associated with lower perception of bitter (PROP) taste in AA individuals (so-called non-tasters or reduced-tasters taking into account that their mean rating intensity for bitter is higher than one but not zero) and a very high perception of bitter (PROP) taste in PP (so-called tasters), PA homozygotes occupying an intermediate position in taste perception. This effect was detected in all the age groups. However, we found a modification of the sex effect by this polymorphism. Therefore, in subjects with the AA genotype, we did not detect significant difference in bitter (PROP) taste perception between men and women. Similar results were previously reported by Khataan, et al. [43] in the multi-ethnic study carried out in the Toronto Nutrigenomics and Health Study analysing healthy men (*n* = 286) and women (*n* = 625) aged 20–29 years. Although the TAS2R38-rs713598 polymorphism is strongly associated with bitter (PROP) taste perception, this is not the only genetic variant associated with bitter (PROP) taste. There is other polymorphism in the same TAS2R28 gene or in other genes in chromosome 7 that have been associated with the bitter (PROP) taste at the genome-wide association study level [70]. Thus, more studies are needed to better understand the genetic influences in bitter taste perception and the homogeneity or heterogeneity by sex in the AA individuals.

For the SCNN1B-rs239345 polymorphism, we found a small statistically significant association with salty taste perception, this association was not modified by age or sex. Very few studies have analysed this polymorphism and the results are still inconsistent [47,49]. Regarding the TAS1R2- rs35874116 polymorphism we did not observe any significant association with sweet taste perception, contributing to the inconsistent findings among studies [46,48,50,51].

It is not only important to characterize the factors that have an influence on different taste perception well but to discover, in the same population, the relationship between taste perception and preferences for each taste. Most studies have directly studied the association between taste perception or its polymorphisms and food intake [40,46,50,51,71] but few have analysed the relationship between taste perception and taste preferences [72,73,74]. In our case, we also analysed taste preferences and, in general, have shown that bitter (PROP) and sour tastes are the least preferred, while salty and sweet taste are the most preferred in agreement with other studies [74,75,76,77]. In addition, we detected some significant differences in taste preference by age and sex. In our study, men liked bitter (PROP) and sour taste more than women but in other population the published results have been heterogeneous [72,78,79,80,81]. Again, age-differences, genetics and study heterogeneity may play a role in these differences. Regarding the association between taste preferences and taste perception, we observed that, in general, that a higher intensity rating for a particular taste was not associated with a higher preference for this taste, in agreement with some studies [74,82]. Interestingly, we observed positive correlations with some of the opposite tastes. Thus, a greater acuity for sweet taste was significantly associated with a higher preference for bitter (PROP) and sour in both men and women. In contrast, the higher perception of the sour taste was associated with higher preference for this taste in women. This is in line with the work of Cornelis, et al. [83] in Canadian adults showing that recalled intensity ratings of the most bitter (PROP) and salty (but not sour foods) were inversely correlated with liking and intake. Likewise, we detected an inverse correlation between salty taste perception and liking in women. This may support some previously reported associations between the lower food intake with bitter (PROP) or salty taste among individuals with higher taste perception for these tastes [47,82,83,84,85]. However, this also illustrates the complexity of the relationships among taste perception-preference and food intake and the need of additional research. In this study, we did not analyse the relationship between taste perception or preferences and food intake, as that would require greater depth and detail of analysis given the multi-dimensionality of diet. This will be tackled in a later study setting off from the new information obtained in the present work.

## 5. Conclusions

In conclusion, in this comprehensive study we have found great variability in the perception of the 5 basic tastes in this population, confirming that increased age is associated with a decrease in the perception of all taste qualities, although mainly in bitter and sour tastes. We detected sex-differences in taste perception; women perceive all the tastes more (except for umami). The TAS2R38-rs713598 polymorphism was strongly associated with bitter (PROP) taste perception and modified the sex-differences for this taste depending on the genotype. Although the relationship between taste perception and preference was complex and differed by taste, we have been able to show that there are certain consistent relationships such the association between higher sweet taste perception and higher preference for bitter (PROP) or sour taste. Although the association between perception, preference and food intake still requires a more detailed analysis, the data obtained in this study may be relevant for precision nutrition, as they support the hypothesis that inter-individual differences in taste perception by age and sex must be taken into account so as to better understand food preferences and food intake and so achieve a greater adherence to dietary recommendations.

## Figures and Tables

**Table 1:** Demographic, clinical and genetic characteristics of the study participants according to sex.

Characteristics	Total (*n* = 1020)	Men (*n* = 365)	Women (*n* = 655)	*p*
Age (years)	43.2 ± 14.3	42.2 ± 14.5	43.8 ± 14.1	0.095
BMI (Kg/m^2^)	27.1 ± 5.3	28.0 ± 5.2	26.6 ± 5.3	<0.001
Obesity cases: *n*, %	281 (27.5)	118 (32.3)	163 (24.1)	0.011
Type 2 diabetes: *n*, %	46 (4.5)	19 (5.2)	27 (4.1)	0.422
Current smokers: *n*, %	206 (20.2)	70 (19.2)	136 (20.8)	0.704
SBP (mmHg)	124.6 ± 17.4	131.4 ± 15.9	120.7 ± 17.1	<0.001
DBP (mmHg)	77.9 ± 10.4	80.6 ± 11.0	76.5 ± 9.8	<0.001
Total cholesterol (mg/dL) ^1^	207.7 ± 39.3	201.9 ± 38.3	210.8 ± 39.5	0.001
HDL-C (mg/dL)	60.2 ± 14.3	52.2 ± 10.8	64.6 ± 14.1	<0.001
LDL-C (mg/dL)	133.6 ± 31.5	133.6 ± 32.1	133.5 ± 31.1	0.947
Triglycerides (mg/dL)	105.5 ± 56.3	118.6 ± 65.5	98.3 ± 49.1	0.138
Fasting glucose (mg/dL)	93.8 ± 19.3	96.6 ± 22.4	92.3 ± 17.2	0.001
Medications: *n*, %				
Antihypertensive drugs	132 (12.9)	67 (18.4)	65 (9.9)	<0.001
Hypolipidaemic drugs	123 (12.1)	52 (14.2)	71 (10.8)	0.098
*TAS2R38*-rs713598: *n*, % ^2^				0.329
Ala/Ala	304 (32.2)	116 (33.9)	188 (31.2)	
Ala/Pro	464 (49.1)	157 (45.9)	307 (50.9)	
Pro/Pro	177 (18.7)	69 (20.2)	108 (17.9)	
*TAS1R2-rs35874116*: *n*, %				0.853
Ile/Ile	345 (36.4)	122 (35.9)	223 (36.6)	
Ile/Val	447 (47.1)	164 (48.2)	283 (46.5)	
Val/Val	157 (16.5)	54 (15.9)	103 (16.9)	
*SCNN1B-rs239345*: *n*, %				0.534
TT	481 (51.9)	169 (51.2)	312 (52.3)	
TA	371 (40.0)	139 (41.8)	233 (39.0)	
AA	75 (8.1)	23 (7.0)	52 (8.7)	

Values are mean ± SD for continuous variables and number (%) for categorical variables; BMI indicates body mass index. SBP indicates Systolic Blood Pressure. DBP indicates Diastolic Blood Pressure; *p*: *p*-value for the comparisons (means or %) between men and women; ^1^: Plasma lipids and fasting glucose were available for 996 participants; ^2^: Valid genotype data were available for 945, 949 and 927 participants, for TAS2R38, TAS1R2 and SCNN1B polymorphisms respectively.

**Table 2:** Association between the perception of different tastants ^1^ and the total taste score in the whole population ^2^.

Taste (Tastant)	Mean (SD) ^3^	Coefficients	Bitter	Sweet	Salty	Sour	Umami
Bitter	2.07 (1.54)	*r*	1				
(PROP 5.5 mM)		*p ^4^*					
Sweet	1.86 (1.14)	*r*	0.162	1			
(Sucrose 400 mM)		*p*	<0.001				
Salty	2.57 (1.35)	*r*	0.255	0.526	1		
(NaCl 200 mM)		*p*	<0.001	<0.001			
Sour	2.73 (1.35)	*r*	0.221	0.433	0.569	1	
(Citric acid 34 mM)		*p*	<0.001	<0.001	<0.001		
Umami	1.98 (1.39)	*r*	0.230	0.359	0.352	0.348	1
(MPG 200 mM)		*p*	<0.001	<0.001	<0.001	<0.001	
Total taste score ^5^	11.22 (4.68)	*r*	0.576	0.664	0.770	0.731	0.658
		*p*	<0.001	<0.001	<0.001	<0.001	<0.001

PROP: 6-n-propylthiouracil.; MPG: L-glutamic acid monopotassium salt monohydrate; ^1^: Five representative tastants for the five tastes (PROP for bitter, sucrose for sweet, NaCl for salty, citric acid for sour and MPG for umami) were tested. Correlation coefficients (Spearman rho) for the intensity rating of the higher concentrations (Concentration V) used are presented; ^2^: *n* = 1020 individuals; ^3^: SD indicates standard deviation; ^4^: *p*-value for the correlation coefficient (*r*: Spearman rho); ^5^: Total taste score: the sum of the scores for the five tastes.

**Table 3:** Association between age (in years) and taste perception in the whole population.

Taste	B ^1^ (SE)	*p* ^1^	B ^2^ (SE)	*p* ^2^	B ^3^ (SE)	*p* ^3^
Bitter (PROP 5.5 mM)	−0.021 (0.003)	3.7 × 10^−10^	−0.020 (0.007)	9.6 × 10^−9^	−0.019 (0.004)	7.8 × 10^−7^
Sweet (Sucrose 400 mM)	−0.012 (0.002)	2.0 × 10^−6^	−0.011 (0.003)	7.0 × 10^−5^	−0.011 (0.003)	1.5 × 10^−4^
Salty (NaCl 200 mM)	−0.013 (0.003)	7.0 × 10^−6^	−0.013 (0.003)	5.8 × 10^−5^	−0.011 (0.003)	1.0 × 10^−3^
Sour (Citric acid 34 mM)	−0.018 (0.003)	1.2 × 10^−9^	−0.016 (0.003)	1.4 × 10^−7^	−0.015 (0.003)	6.0 × 10^−6^
Umami (MPG 200 mM)	−0.013 (0.003)	1.5 × 10^−5^	−0.012 (0.003)	3.0 × 10^−4^	−0.013 (0.004)	2.5 × 10^−4^
Total taste score ^4^	−0.077 (0.010)	2.9 × 10^−14^	−0.071 (0.011)	2.1 × 10^−11^	−0.070 (0.029)	2.4 × 10^−9^

Values are regression coefficients (B), standard errors (SE), both expressed in points per year. (*n* = 1020 participants); Tastants for each taste were tested at the maximum concentration analysed (Category 5); 1: *p*-value for the model 1 unadjusted; ^2^: *p*-value for the model 2 adjusted for sex and body mass index (BMI); ^3^: *p*-value for the model 3 additionally adjusted for diabetes, smoking and medications (lipid-lowering and antihypertensive drugs); ^4^: Total taste score: the sum of the scores for the five tastes.

**Table 4:** Association between sex and taste perception in the whole population.

Taste	B (SE)	*p* ^1^	*p*^2^ Interaction Term (Sex × Age)
Bitter (PROP 5.5 mM)	0.472 (0.100)	1.3 × 10^−6^	0.965
Sweet (Sucrose 400 mM)	0.166 (0.076)	0.026	0.172
Salty (NaCl 200 mM)	0.417 (0.089)	1.3 × 10^−6^	0.410
Sour (Citric acid 34 mM)	0.498 (0.087)	1.6 × 10^−8^	0.703
Umami (MPG 200 mM)	0.169 (0.093)	0.069	0.107
Total taste score ^3^	1.720 (0.301)	1.4 × 10^−8^	0.324

Values are regression coefficients (B) and standard errors (SE), obtained for women in comparison with men (reference) expressed in points for the corresponding taste. Models were additionally adjusted for age, body mass index (BMI), smoking and medications (lipid-lowering and antihypertensive drugs) (model 3). *n* = 365 men and 655 women; Tastants for each taste were tested at the maximum concentration analysed (category 5); ^1^: *p*-value obtained for the sex variable in the multivariate regression model (model 3) without interaction; ^2^: *p*-value for the additionally tested interaction term sex and age in the multivariate adjusted model 3; ^3^: Total taste score: the sum of the scores for the five tastes.

**Table 5:** Association between the preference of different tastes ^1^ in the whole population ^2^ and by sex.

Taste	Mean (SD) ^3^	Coefficients	Bitter Taste Preference	Sweet Taste Preference	Salty Taste Preference	Sour Taste Preference
	**Whole population**					
Bitter taste	0.60 (0.80)	*r*	1			
preference		*p* ^4^				
Sweet taste	2.43 (0.80)	*r*	−0.175	1		
preference		*p*	<0.001			
Salty taste	2.22 (0.91)	*r*	0.048	−0.053	1	
preference		*p*	0.139	0.100		
Sour taste	0.63 (0.84)	*r*	0.346	−0.125	0.131	1
preference		*p*	<0.001	<0.001	<0.001	
	**Men**					
Bitter taste	0.75 (0.87) ^5^	*r*	1			
preference		*p*				
Sweet taste	2.45 (0.74)	*r*	−0.181	1		
preference		*p*	<0.001			
Salty taste	2.23 (0.86)	*r*	0.068	−0.027	1	
preference		*p*	0.216	0.622		
Sour taste	0.70 (0.81) ^6^	*r*	0.430	−0.039	0.199	1
preference		*p*	<0.001	0.471	<0.001	
	**Women**					
Bitter taste	0.51 (0.75) ^5^	*r*	1			
preference		*p*				
Sweet taste	2.42 (0.83)	*r*	−0.173	1		
preference		*p*	<0.001			
Salty taste	2.22 (0.94)	*r*	0.040	−0.067	1	
preference		*p*	0.318	0.096		
Sour taste	0.60 (0.86) ^6^	*r*	0.286	−0.167	0.100	1
preference		*p*	<0.001	<0.001	0.013	

^1^: Preference for bitter (PROP), sweet, salty and sour tastes was assessed by questionnaire. Responses ranked from cero to three for all the tastants; ^2^: Taste preference data were available from 955 participants (*n* = 339 men and 616 women); ^3^: SD indicates standard deviation; ^4^: *p*-value for the correlation coefficient (*r*: Spearman rho) in the whole population and by sex; ^5^: Taste perception significantly different between men and women (*p* < 0.001 Mann-Whitney test); ^6^: Taste perception significantly different between men and women (*p* = 0.007 Mann-Whitney test).

**Table 6:** Association between the perception of different tastants^1^ and the taste preference by sex ^2^.

Taste (Tastant)	Sex	Coeffi-Cients	Bitter Taste Preference	Sweet Taste Preference	Salty Taste Preference	Sour Taste Preference
	**Men**					
Bitter		*r*	0.010	−0.040	−0.020	0.035
(PROP 5.5 mM)		*p* ^3^	0.858	0.461	0.712	0.521
Sweet		*r*	0.111	−0.043	0.044	0.126
(Sucrose 400 mM)		*p*	0.042	0.428	0.415	0.021
Salty		*r*	0.114	0.016	0.011	0.090
(NaCl 200 mM)		*p*	0.036	0.774	0.839	0.098
Sour		*r*	0.090	−0.030	0.018	0.066
(Citric acid 34 mM)		*p*	0.099	0.582	0.742	0.225
Umami		*r*	0.178	−0.060	−0.050	0.135
(MPG 200 mM)		*p*	0.001	0.269	0.362	0.013
	**Women**					
Bitter		*r*	0.026	0.059	−0.011	0.002
(PROP 5.5 mM)		*P* ^3^	0.515	0.140	0.789	0.951
Sweet		*r*	0.110	0.038	−0.011	0.101
(Sucrose 400 mM)		*p*	0.006	0.352	0.783	0.012
Salty		*r*	0.037	0.062	−0.103	0.040
(NaCl 200 mM)		*p*	0.359	0.126	0.011	0.325
Sour		*r*	0.080	0.015	−0.039	0.085
(Citric acid 34 mM)		*p*	0.046	0.706	0.335	0.036
Umami		*r*	0.098	0.008	−0.066	0.015
(MPG 200 mM)		*p*	0.015	0.846	0.103	0.702

PROP: 6-n-propylthiouracil; MPG: L-glutamic acid monopotassium salt monohydrate; ^1^: Five representative tastants for the five tastes (PROP for bitter, sucrose for sweet, NaCl for salty, citric acid for sour and MPG for umami) were tested. Correlation coefficients (Spearman rho) for the intensity rating of the higher concentrations (Concentration V) used are presented. Taste preference was measured by questionnaire; ^2^: With taste preference data (*n* = 339 men and 616 women); ^3^: *p*-value for the correlation coefficient (*r*: Spearman rho).

## Supplementary Materials

The following are available online at http://www.mdpi.com/2072-6643/10/10/1539/s1, Table S1: Association between the perception of different tastants by sex, Table S2: Association between the preference of different tastes1 by age groups2, Table S3: Association between the perception of different tastants1 and the taste preference in the whole population2, Figure S1: Ratings of perceived taste intensity in response to five detailed concentrations (I to V) of tastants, Figure S2: Total taste score (sum of intensity ratings of the five tastes) (points) in the whole population (*n* = 1020) for the tastants at the maximum concentration tested, Figure S3: Taste perception (intensity rating) of bitter (PROP, 5.5 mM) and umami (MPG 200Mm) at concentration V by sex and age groups and Figure S4: Salty taste perception (intensity rating of NaCl, 200 mM) by the SCNN1B-rs239345 polymorphism for the recessive model in the whole population, in men and women and by age groups.
