# Supplementary material for: Bitter, Sweet, Salty, Sour and Umami Taste Perception Decreases with Age: Sex-Specific Analysis, Modulation by Genetic Variants and Taste-Preference Associations in 18 to 80 Year-Old Subjects
Source: Nutrients. 2018 Oct 18;10(10):1539. doi: 10.3390/nu10101539 (PMC6213100; doi:10.3390/nu10101539)

ONLINE SUPPLEMENTARY MATERIAL

Bitter, Sweet, Salty, Sour and Umami Taste Perception Decreases with Age: Sex-Specific Analysis, Modulation by Genetic Variants and Taste-Preference Associations in 18 to 80 Year-Old Subjects

Rocio Barragán, Oscar Coltell, Olga Portolés, Eva M. Asensio, José V. Sorlí, Carolina Ortega-Azorín, José I González, Carmen Sáiz, Rebeca Fernández-Carrión, Jose M. Ordovás, Dolores Corella

Supplementary Figure S1 2
Supplementary Table S1 3
Supplementary Figure S2 5
Supplementary Figure S3 6
Supplementary Table S2 7
Supplementary Table S3 9
Supplementary Figure S4 10

Supplementary Figure S1: Ratings of perceived taste intensity in response to five detailed concentrations (I to V) of tastants for sweet, salty, sour, and umami (n = 1019, 1016, 1014, 1016 and 1020 for the concentrations I, II, III, IV and V, respectively) in the whole population. Values are means and error bars are standard error of means.

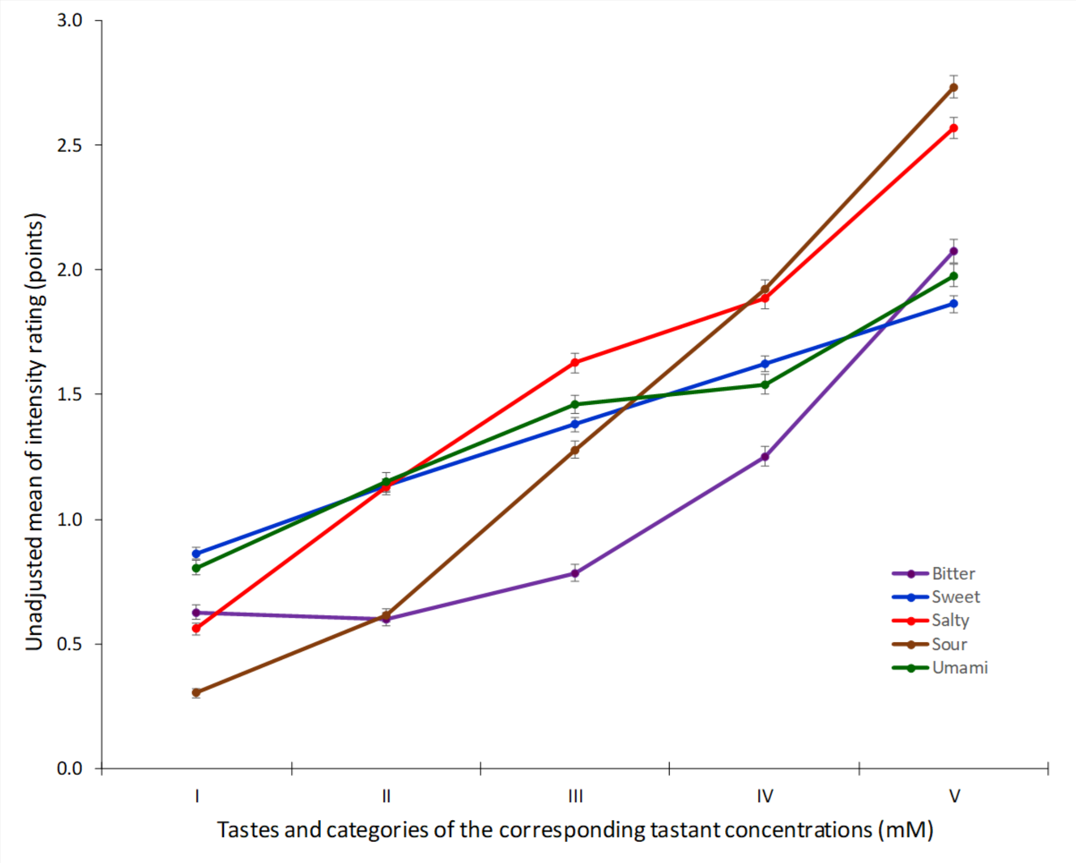

Taste	Tastant	Conc. I (mM)	Conc. II (mM)	Conc. III (mM)	Conc. IV (mM)	Conc. V (mM)
Bitter	PROP	0.055	0.17	0.55	1.7	5.5
Sour	Citric acid	1	5	10	17	34
Salty	NaCl	25	50	75	100	200
Sweet	Sucrose	100	150	200	300	400
Umami	Glutamate	25	50	75	100	200

Supplementary Table S1: Association between the perception of different tastants by sex.

Taste (tastant)	Sex	Mean (SD)[3]		Bitter	Sweet	Salty	Sour	Umami
	**Men**							
Bitter (PROP 5.5 mM)		1.79 (1.44)	*r*	1				
			p[4]					
Sweet (Sucrose 400 mM)		1.76 (1.04)	*r*	0.108	1			
			p	0.039				
Salty (NaCl 200 mM)		2.30 (1.32)	*r*	0.253	0.483	1		
			p	<0.001	<0.001			
Sour (Citric acid 34 mM)		2.41 (1.33)	*r*	0.192	0.379	0.536	1	
			p	<0.001	<0.001	<0.001		
Umami (MPG 200 mM)		1.88 (1.38)	*r*	0.142	0.370	0.354	0.386	1
			p	0.006	<0.001	<0.001	<0.001	
Total taste score[5]		10.14 (4.42)	*r*	0.521	0.632	0.762	0.729	0.679
			p	<0.001	<0.001	<0.001	<0.001	<0.001
	**Women**							
Bitter (PROP 5.5 mM)		2.24 (1.57)	*r*	1				
			p					
Sweet (Sucrose 400 mM)		1.92 (1.19)	*r*	0.183	1			
			p	<0.001				
Salty (NaCl 200 mM)		2.72 (1.35)	*r*	0.234	0.547	1		
			p	<0.001	<0.001			
Sour (Citric acid 34 mM)		2.91 (1.33)	*r*	0.201	0.459	0.571	1	
			p	<0.001	<0.001	<0.001		
Umami (MPG 200 mM)		2.03 (1.40)	*r*	0.269	0.351	0.349	0.323	1
			p	<0.001	<0.001	<0.001	<0.001	
Total taste score		11.02 (4.74)	*r*	0.586	0.685	0.766	0.719	0.652
			p	<0.001	<0.001	<0.001	<0.001	<0.001

PROP: 6-n-propylthiouracil.

MPG: L-glutamic acid monopotassium salt monohydrate.

[1]: Five representative tastants for the five tastes (PROP for bitter, sucrose for sweet, NaCl for salty, citric acid for sour and MPG for umami) were tested (Concentration V). Correlation coefficients (Spearman rho) for the intensity rating of the higher concentrations (Category V) used are presented.

[2]: n = 1020 individuals (n = 365 men and 655 women).

[3]: SD indicates standard deviation.

[4]: *P-value* for the correlation coefficient (r: Spearman rho).

[5]: Total taste score: the sum of the scores for the five tastes.

Supplementary Figure S2: Total taste score (sum of intensity ratings of the five tastes) (points) in the whole population (n =1020) for the tastants at the maximum concentration tested (concentration V: PROP 5.5 mM; sucrose 400 mM, NaCl 200 mM; citric acid 34 mM; and MPG 200 mM).

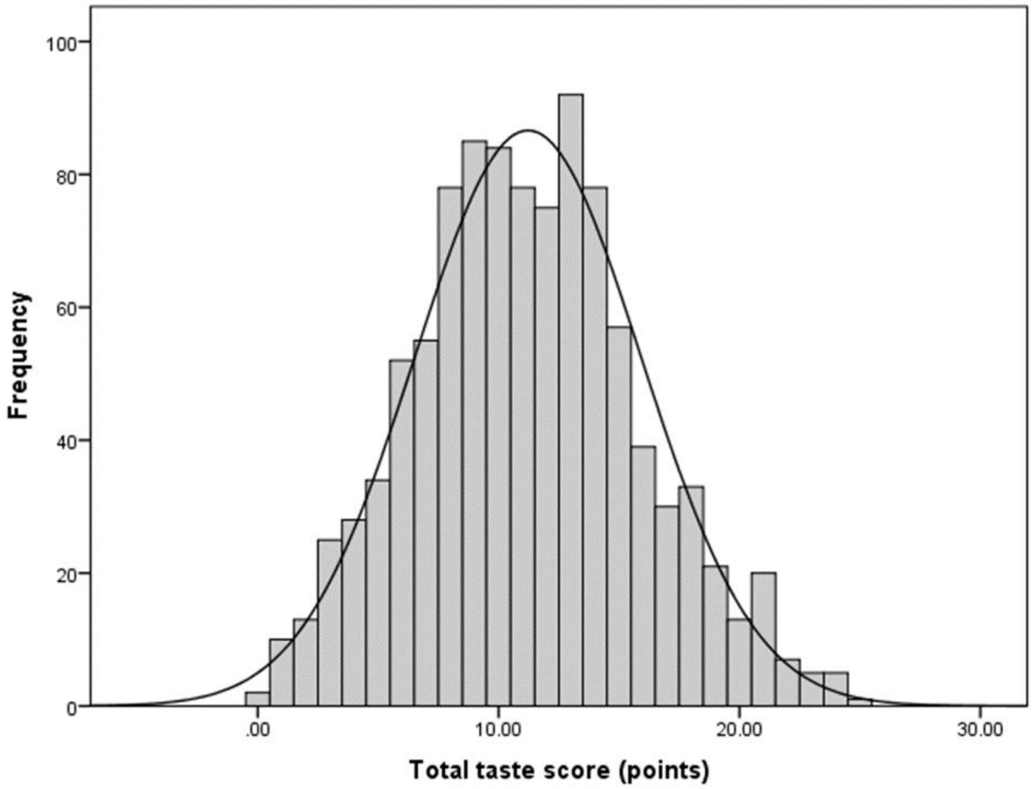

Supplementary Figure S3: Taste perception (intensity rating) of bitter (PROP 5.5 mM) and umami (MPG 200Mm) at concentration V by sex (n = 365 men and 655 women) and age groups [18-36 years (n = 342), 37-50 years (n = 329), and 51-80 years (n = 349)]. Means were adjusted for sex, age, diabetes, body mass index, smoking and medications (model 3). *P-values* show the statistical significance for the sex variable per age groups in the multivariate adjusted model 3. The P-value for interaction terms between sex and age for bitter and umami was additionally tested in their corresponding models 3. Error bars are SE.

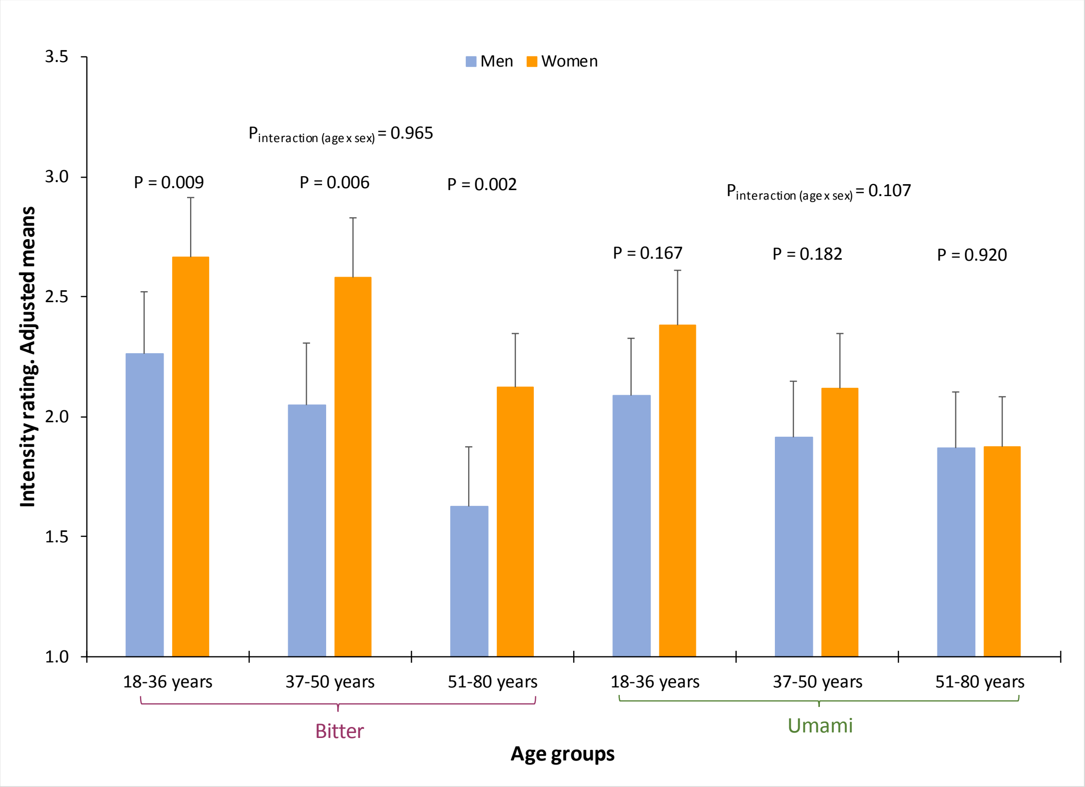

Supplementary Table S2: Association between the preference of different tastes[1] by age groups[2].

Taste	Mean (SD)[3]		Bitter taste preference	Sweet taste preference	Salty taste preference	Sour taste preference
	**18-36 years**					
Bitter taste preference	0.72 (0.82)[5]	*r*	1			
		p[4]				
Sweet taste preference	2.55 (0.66)[6]	*r*	-0.170	1		
		p	0.002			
Salty taste preference	2.30 (0.84)	*r*	-0.037	-0.089	1	
		p	0.511	0.115		
Sour taste preference	0.61 (0.77)	*r*	0.393	-0.145	0.111	1
		p	<0.001	0.009	0.048	
	**37-50 years**					
Bitter taste preference	0.56 (0.76)[5]	*r*	1			
		p				
Sweet taste preference	2.43 (0.83)[6]	*r*	-0.135	1		
		p	0.017			
Salty taste preference	2.22 (0.94)	*r*	0.121	-0.141	1	
		p	0.033	0.013		
Sour taste preference	0.62 (0.85)	*r*	0.303	-0.126	0.167	1
		p	<0.001	0.025	0.003	
	**51-80 years**					
Bitter taste preference	0.51 (0.80)[5]	*r*	1			
		p				
Sweet taste preference	2.31 (0.88)[6]	*r*	-0.263	1		
		p	<0.001			
Salty taste preference	2.16 (0.96)	*r*	0.039	0.034	1	
		p	0.486	0.549		
Sour taste preference	0.67 (0.90)	*r*	0.347	-0.102	0.122	1
		p	<0.001	0.069	0.029	

[1]: Preference for bitter, sweet, salty and sour tastes was assessed by questionnaire. Responses ranked from zero to three for all the tastants.

[2]: Taste preference data were available from 955 participants (n = 318 in the group aged 18-36 years; 313 in the group aged 37-50 years; and 324 in the group aged 51-80 years).

[3]: SD indicates standard deviation.

[4]: *P-value* for the correlation coefficient (r: Spearman rho) by age groups.

[5]: Taste perception significantly different among age groups ($p < 0.001$ Kruskal-Wallis test)

[6]: Taste perception significantly different among age groups ($p = 0.004$ Kruskal-Wallis test)

Supplementary Table S3: Association between the perception of different tastants[1] and the taste preference in the whole population[2].

Taste (tastant)		Bitter taste preference	Sweet taste preference	Salty taste preference	Sour taste preference
Bitter	*r*	0.001	0.025	-0.012	8.4×10^{-4}
(PROP 5.5 mM)	*p*[3]	0.972	0.434	0.701	0.979
Sweet	*r*	0.101	0.012	0.009	0.102
(Sucrose 400 mM)	*p*	0.002	0.722	0.777	0.002
Salty	*r*	0.043	0.045	-0.059	0.040
(NaCl 200 mM)	*p*	0.189	0.167	0.066	0.214
Sour	*r*	0.055	0.002	-0.015	0.058
(Citric acid 34 mM)	*p*	0.088	0.953	0.653	0.075
Umami	*r*	0.119	-0.016	-0.059	0.051
(MPG 200 mM)	*p*	<0.001	0.631	0.069	0.113

PROP: 6-n-propylthiouracil.

MPG: L-glutamic acid monopotassium salt monohydrate.

[1]: Five representative tastants for the five tastes (PROP for bitter, sucrose for sweet, NaCl for salty, citric acid for sour and MPG for umami) were tested. Correlation coefficients (Spearman rho) for the intensity rating of the higher concentrations (Concentration V) used are presented.

[2]: n = 1020 individuals.

[3]: *P-value* for the correlation coefficient (r: Spearman rho).

Supplementary Figure S4: Salty taste perception (intensity rating of NaCl, 200 mM) (n = 927) by the SCNN1B-rs239345 polymorphism for the recessive model (n = 481 for TT, n = 371 for TA, and n = 75 for AA) in the whole population, in men and women and by age groups. Means were adjusted for sex, age, diabetes, body mass index, smoking and medications (model 3). P-values show the statistical significance of the SCNN1B-rs239345 polymorphism, in the whole population, as well as the interaction terms between the SCNN1B-rs239345 polymorphism and sex and the SCNN1B-rs239345 polymorphism and age. All P-values were multivariate adjusted (model 3). The P-value for interaction term between the TAS2R38-rs713598 and age groups was additionally tested in model 3. Error bars are SE.

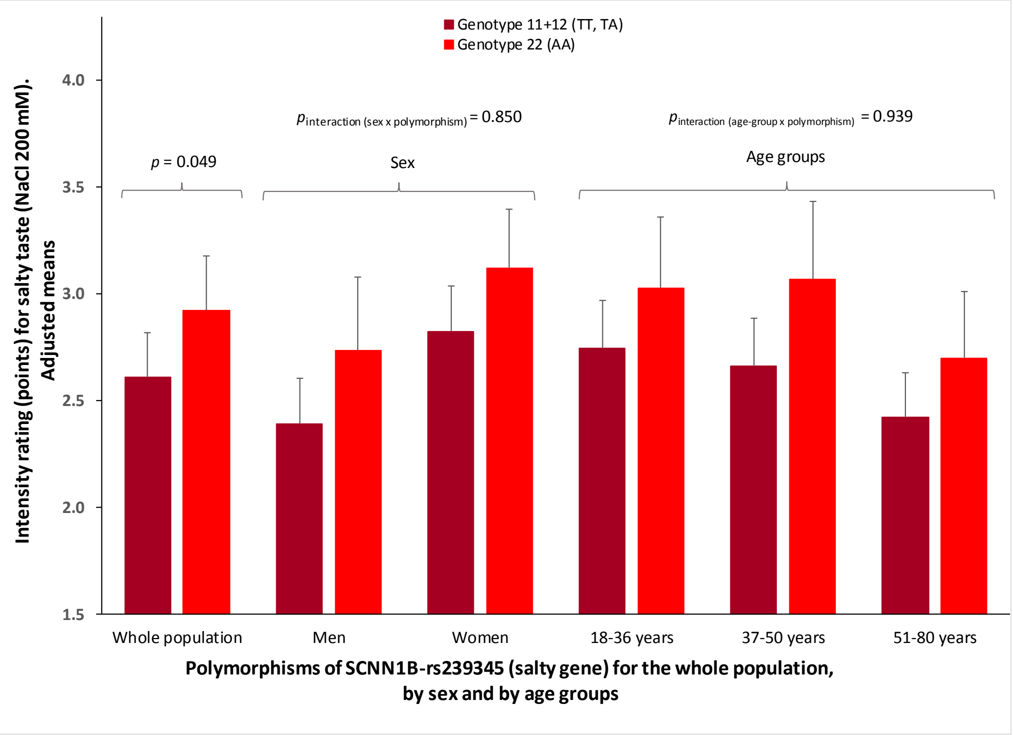

Supplement: Supplementary file 1 [file nutrients-10-01539-s001.pdf]
